# Antimicrobial Resistance of Bacteria Isolated From Patients With Cancer

**DOI:** 10.7759/cureus.116860

**Published:** 2026-09-24

**Authors:** Yetzil Valente-Morales, Sandra Quintana-Ponce, Margarita Y Kuri-Guinto, Sacnite D Díaz-Gonzaléz, Rocio G Hernández-Nava, Nicolás R Barlandas-Rendón

**Affiliations:** 1 Clinical Microbiology, Faculty of Natural Sciences, Autonomous University of Guerrero, Chilpancingo de los Bravo, MEX; 2 Clinical Oncology, State Cancerology Institute “Dr. Arturo Beltrán Ortega”, Acapulco de Juarez, MEX; 3 Nutrition, School of Nutrition, Autonomous University of Guerrero, Acapulco de Juarez, MEX; 4 Laboratory Medicine, Faculty of Natural Sciences, Autonomous University of Guerrero, Chilpancingo de los Bravo, MEX

**Keywords:** antimicrobial resistance, broad-spectrum beta-lactamases, cancer patients, enterobacteria, gram-negative bacteria, multidrug resistance

## Abstract

Background: Cancer patients are at high risk of developing infections caused by multidrug-resistant (MDR) microorganisms. Monitoring antimicrobial resistance (AMR) is essential for guiding treatment in this vulnerable population. This study aimed to evaluate AMR patterns and temporal evolution among clinical bacterial isolates-comparing pre-pandemic (2016-2019) versus pandemic (2020-2021) periods and care settings (outpatient vs. inpatient)-recovered from cancer patients at a tertiary care center in Guerrero, Mexico.

Materials and methods: An observational, cross-sectional, retrospective study was conducted between 2016 and 2021. A total of 1,019 strains were analyzed using WHONET 2023 software, following the Clinical and Laboratory Standards Institute (CLSI) M100 cut-off values. Categorical variables were compared using Pearson’s chi-squared test (χ²). For antibiotics with absolute frequencies less than 5, Fisher’s exact test was applied using STATA statistical software version 14.2 (StataCorp LLC, College Station, TX, USA).

Results: Enterobacteriaceae accounted for 73.1% of the isolates, with *Escherichia coli* (42.3%, n = 431) and *Klebsiella pneumoniae* (14.0%, n = 143) being the predominant strains. The overall prevalence of extended-spectrum beta-lactamases (ESBLs) was 22.6%. In *E. coli*, an increase in resistance was observed starting in 2019, with resistance rates > 80.0% for ampicillin and cefixime. When comparing the 2020 period with 2021, resistance in *E. coli* increased significantly for ciprofloxacin (χ² = 4.144, p = 0.042) and ceftriaxone (χ² = 4.100, p = 0.043). Regarding MDR, 51.0% of *E. coli* isolates were MDR, while 32.2% of *K. pneumoniae* strains exhibited extensive drug resistance (XDR). In addition, possible pandrug-resistant (PDR) phenotypes were identified in 14.3% of *Acinetobacter* spp. isolates. Isolates from hospitalized patients showed higher resistance rates compared to those from outpatients with regard to *E. coli* for ampicillin/sulbactam (χ² = 4.422, p = 0.035), piperacillin/tazobactam (χ² = 17.519, p < 0.001), and cefazolin (χ² = 6.746, p = 0.009).

Conclusions: The results demonstrate a high predominance of Enterobacteriaceae among clinical bacterial isolates from the evaluated oncology population. *E. coli* exhibited the highest proportion of MDR phenotypes (51.0%), whereas *K. pneumoniae* showed the highest frequency of XDR (32.2%). The high level of resistance observed among isolates from hospitalized patients could help guide empirical treatment toward alternative therapeutic options.

## Introduction

Cancer is defined as a group of diseases characterized by uncontrolled cell proliferation and the ability to invade adjacent tissues or organs through metastasis [1]. Mortality rates from malignant tumors have shown a steady increase, rising from 6.09 to 7.06 deaths per 10,000 inhabitants between 2010 and 2021. This trend persists, as cancer was the third leading cause of death in Mexico from January to September 2025, with a total of 69,172 recorded deaths [2,3].

Prolonged and severe neutropenia is one of the most significant risk factors for sepsis and mortality in cancer patients. The most common clinical presentations or sources of infection include mucositis and esophagitis (40%-76%), intra-abdominal infections (15%-50%), gastrointestinal tract infections (30%), central venous catheter-associated infections (5%-26%), and respiratory tract infections (0.4%-35%) [4-8].

Antimicrobial resistance (AMR) is a phenomenon of selective pressure that occurs in bacteria; in recent years, this process has accelerated, becoming a global health threat reflected in increased infection rates and longer hospital stays [9]. In 2017, the World Health Organization (WHO) published its first list of priority antibiotic-resistant pathogens, which was subsequently updated in May 2024 [10,11].

Reports from 2022 indicate resistance rates exceeding 50.0% in pathogens causing hospital-acquired sepsis, such as *Klebsiella pneumoniae* and *Acinetobacter* spp. Notably, 8.0% of these *K. pneumoniae* isolates are already resistant to carbapenems, drugs considered a last resort. Furthermore, *Escherichia coli* has transitioned from being a natural commensal to the leading cause of urinary tract infections (UTIs) in cancer patients, followed by *K. pneumoniae* [12-14]. Additionally, *Pseudomonas aeruginosa* has been detected in isolates from adult and pediatric cancer patients, exhibiting high resistance to carbapenems (55.6%), particularly to imipenem (IMP) and meropenem (MEM) [15,16]. Additionally, the presence of Gram-positive bacteria has been reported in oncology patients, with *Staphylococcus aureus* being the predominant pathogen [15,17].

Meanwhile, efforts are underway in Mexico to address bacterial resistance; on June 5, 2018, an agreement was published in the Official Gazette of the Federation (OGF) declaring the mandatory status of the National Action Strategy against Antimicrobial Resistance [18]. In light of the above, the primary objective of this study was to evaluate the overall prevalence of AMR among key bacterial pathogens isolated from cancer patients at a tertiary care oncology center. As secondary objectives, we aimed to compare resistance profiles between the pre-pandemic (2016-2019) and post-pandemic (2020-2021) periods, and evaluate differences in resistance rates between inpatient and outpatient clinical settings.

## Materials and methods

Study design

A retrospective, observational study was conducted to analyze the prevalence and evolution of AMR in bacterial isolates from patients treated at a regional referral center during the period from January 2016 to December 2021. The data were divided into two comparative periods: the pre-pandemic period (2016-2019) and the pandemic period (2020-2021).

Study population and selection strategy

The study included samples collected from urine (79.3%, n = 808), wounds/exudates/soft tissue (13.3%, n = 136), and bloodstream (2.5%, n = 26) from cancer patients of all ages and both sexes who were admitted to or treated at the regional referral center between January 2016 and December 2021. Stool specimens or those of unknown origin (4.8%, n = 49) were excluded. Throughout this study, the clinical bacterial isolate served as the primary unit of analysis, and only the first isolate per patient per calendar year was considered. Isolates obtained from patients with incomplete medical histories and those for which susceptibility testing was not performed were excluded.

Microbiological processing and identification

Clinical samples from blood, urine, secretions, exudates, and wounds were inoculated onto selective and non-selective culture media (blood agar, MacConkey agar, mannitol salt agar, and Mueller-Hinton agar) in accordance with the institution's standard operating procedures. Bacterial identification and antimicrobial susceptibility testing (AST) were performed using the VITEK 2 Compact system (bioMérieux, France). The system uses plastic cards containing microwells with specific substrates for bacterial identification or antimicrobial agents for susceptibility testing. Species identification was achieved through automated colorimetric analysis of metabolic and biochemical profiles, whereas antimicrobial susceptibility was determined by monitoring bacterial growth to establish minimum inhibitory concentrations (MICs) according to standardized breakpoints.

AST data were digitally exported from the VITEK® 2 Compact system and converted into WHONET 2023 format using the BacLink tool. Isolate validation was automatically performed using the VITEK® 2 System, and the data were subsequently processed in WHONET 2023 according to Clinical and Laboratory Standards Institute (CLSI) M100 criteria [19]. Duplicate data were removed directly in WHONET, retaining only the first isolate per patient per calendar year. Statistical analysis was subsequently performed using STATA 14.2 (StataCorp LLC, College Station, TX, USA).

For taxonomic identification at the species level, VITEK® 2 GN cards (for Gram-negative bacilli) and VITEK® 2 GP cards (for Gram-positive cocci) were used. Likewise, antimicrobial susceptibility profiles and MIC determinations were evaluated using the VITEK® 2 AST-N and AST-P series cards during the study period. Quantitative MIC values for all isolates (2016-2021) were preserved in the primary database. To eliminate temporal trends resulting from changing interpretive criteria across different annual editions, susceptibility categories (susceptible, intermediate, or resistant) were interpreted across the entire multiyear dataset using the reference criteria established in the CLSI M100 33rd Edition (2023) standards [19].

AST was performed for amikacin (AMK), ampicillin (AMP), AMP/sulbactam (SAM), aztreonam (ATM), cefepime (FEP), cefazolin (CZO), cefixime (CFM), ceftriaxone (CRO), ceftazidime (CAZ), cefotaxime (CTX), cefuroxime (CXM), ciprofloxacin (CIP), norfloxacin (NOR), ertapenem (ETP), gentamicin (GEN), MEM, nitrofurantoin (NIT), trimethoprim/sulfamethoxazole (SXT), tetracycline (TCY), tobramycin (TOB), piperacillin/tazobactam (TZP), and fosfomycin (FOS).

Isolates were classified directly within WHONET software as multidrug-resistant (MDR) if they exhibited resistance to at least one agent in three or more antimicrobial categories. Extensive drug resistance (XDR) was defined as resistance to at least one agent in all but two or fewer antimicrobial categories (i.e., remaining susceptible to only one or two categories), whereas possible pandrug resistance (PDR) was defined as resistance to all agents in all evaluated antimicrobial categories via WHONET 2023, in accordance with the international expert consensus definitions from the European Centre for Disease Prevention and Control (ECDC) and the Centers for Disease Control and Prevention (CDC) [20].

Ethical considerations

To conduct this research, authorization was obtained from the Ethics Committee of the State Oncology Institute in the city of Acapulco de Juarez, Mexico. Throughout the research process, the health and safety measures established in Chapter 1 of Title II, "Ethical Aspects of Research Involving Human Subjects," of the Regulations of the General Health Law regarding health research were taken into account [21]. Due to the retrospective and observational nature of the study, as well as the use of anonymized laboratory data, the Ethics Committee waived the requirement for informed consent, approving the research under protocol number DGIECAN/CCHGC/CEI/PRO-017-2021. In strict compliance with institutional ethics guidelines and national data protection regulations (General Law on Protection of Personal Data Held by Obligated Entities), the data were analyzed as de-identified routine surveillance records.

Statistical analysis

For the evaluation of AMR, two complementary approaches were used: descriptive chronological analysis and the comparison of proportions. The chronological distribution of resistance was assessed through an annual descriptive evaluation of absolute frequencies and percentages to analyze the behavior of resistant isolates over the study years. For the comparative analysis of resistance rates between the pre-pandemic (2016-2019) and pandemic (2020-2021) periods, as well as between inpatient and outpatient settings, a comparison of proportions based on 2 × 2 contingency tables was applied. Categorical variables were compared using Pearson’s chi-squared test (χ²) with one degree of freedom (df = 1), executed using STATA statistical software version 14.2. For antibiotics with absolute frequencies < 5, Fisher’s exact test was applied instead. To evaluate longitudinal resistance trends across the study period (2016-2021) for *E. coli*, the Cochran-Armitage test for linear trend (chi-squared test for trend) was performed, with the slope indicating the annual rate and direction of change in resistance proportions. In all cases, a probability level of p < 0.05 was established as the threshold for statistical significance.

## Results

Distribution and prevalence of bacterial isolates

During the 2016-2021 period, a total of 1,019 bacterial isolates were obtained and analyzed. The samples primarily originated from female patients (64.1%). Regarding the healthcare setting, 88.1% (n = 898) of the isolates were obtained from the outpatient service, whereas 11.9% (n = 121) originated from the hospitalization unit. The age distribution showed a predominance of adults (85.9%, n = 875) in the overall sample, a pattern that was also reflected in the outpatient service. Conversely, children and adolescents predominated in the hospitalization ward (62.8%, n = 76) (Table 1).

**Table 1:** Distribution of bacterial isolates by age group and service area. Values are expressed as absolute count n and within-group percentage (%). Denominators for percentage calculations correspond to the total isolates in each specific healthcare setting column (n).

Age group	Outpatient care, n (%)	Hospitalization, n (%)	Total cohort, n (%)
Children and adolescents (ages 0 to 17)	61 (6.8%)	76 (62.8%)	137 (13.4%)
Young adults (ages 18 to 25)	7 (0.8%)	0 (0%)	7 (0.7%)
Adults (≥26 years)	830 (92.4%)	45 (37.2%)	875 (85.9%)
Total isolates	898 (100%)	121 (100%)	1,019 (100%)

Evolutionary profile and resistance patterns in Gram-negative and Gram-positive bacteria

The most frequently isolated group of bacteria was Enterobacteriaceae (73.1%), followed by Gram-positive cocci (16.6%) and non-fermenting Gram-negative bacilli (6.5%). *E. coli* was the most frequently isolated bacterium (42.3%), followed by *K. pneumoniae* (14.0%) and *P. aeruginosa* (4.3%).

A prevalence of 22.6% of extended-spectrum beta-lactamase (ESBL)-producing bacteria was identified; *E. coli* accounted for 80.0% and *K. pneumoniae* for 20.0%. *E. coli* maintained the highest prevalence throughout the entire study period, reaching its peak number of isolates in 2017 (n = 150). In contrast, *K. pneumoniae* showed a progressive increase in its relative proportion over time, with a marked rise observed in 2021.

Among the Gram-positive isolates, *Staphylococcus* species were classified into two groups due to their low frequency: coagulase-positive (including *S. aureus* and *Staphylococcus pseudintermedius*) and coagulase-negative (*Staphylococcus haemolyticus*, *Staphylococcus epidermidis*, *Staphylococcus saprophyticus*, *Staphylococcus hominis*, *Staphylococcus lugdunensis*, and *Staphylococcus warneri*). The remaining isolated bacteria were grouped as "other microorganisms" (29.3%) (Figure 1).

**Figure 1:**
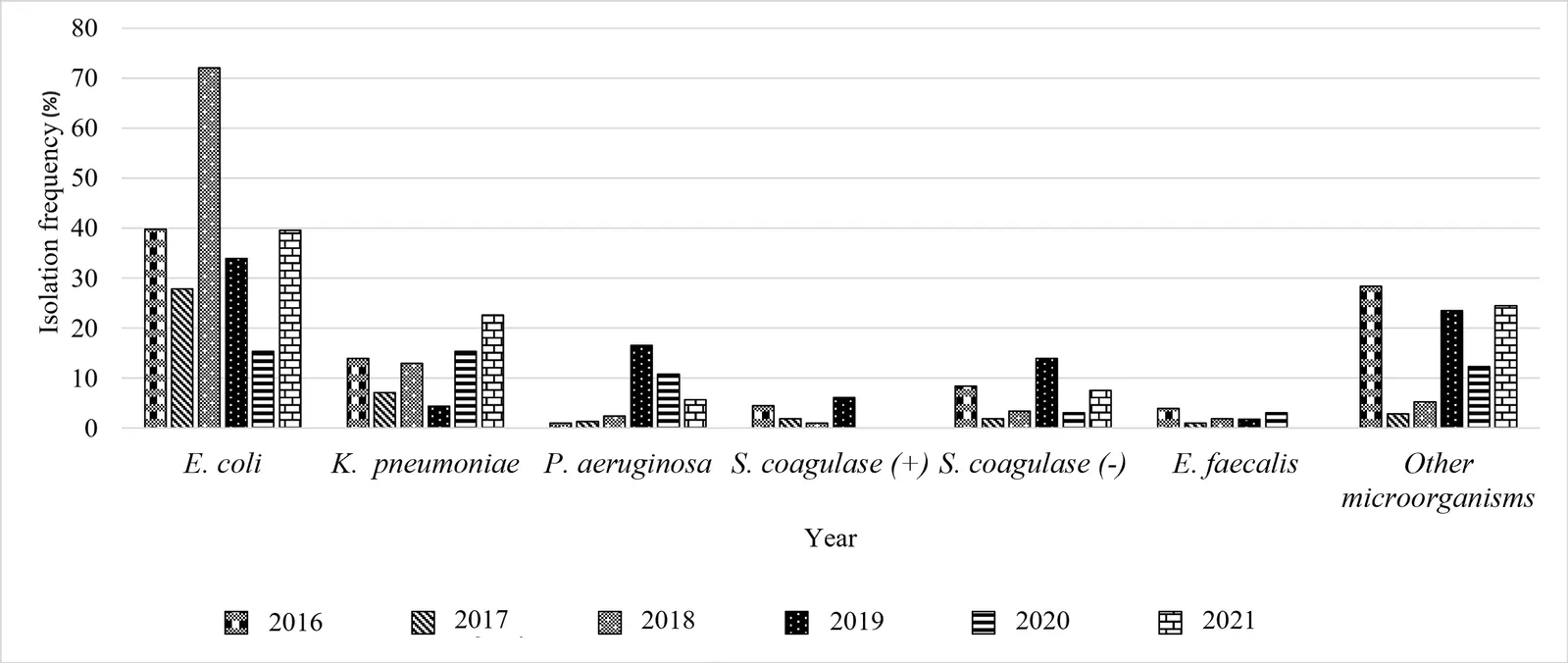
Chronological distribution of AMR of the most common bacterial isolates from 2016 to 2021 in State Cancerology Institute. AMR: antimicrobial resistance

An analysis of the evolution of AMR in *E. coli* was performed, revealing that CAZ showed an upward trend, reaching 37.0% in 2021, while CRO rose above 60.0%, and FEP remained between 20.0% and 30.0% resistance. It is worth noting that CFM was not evaluated from 2016 to 2018; however, following its incorporation in 2019, it showed AMR levels above 88.9%, recording its maximum value (100%) in the first year of its determination. Meanwhile, AMP reached 88.9%, and at the end of the period, SAM was observed above 30.0%. Regarding fluoroquinolones, CIP showed AMR levels above 70.0% in 2016; starting in 2017, resistance decreased progressively to 47.2% in 2020, but increased to 73.1% in 2021. A comparison between 2020 and 2021 using Pearson's chi-squared test revealed significant differences (χ² = 4.14, p = 0.042) (Figure 2).

**Figure 2:**
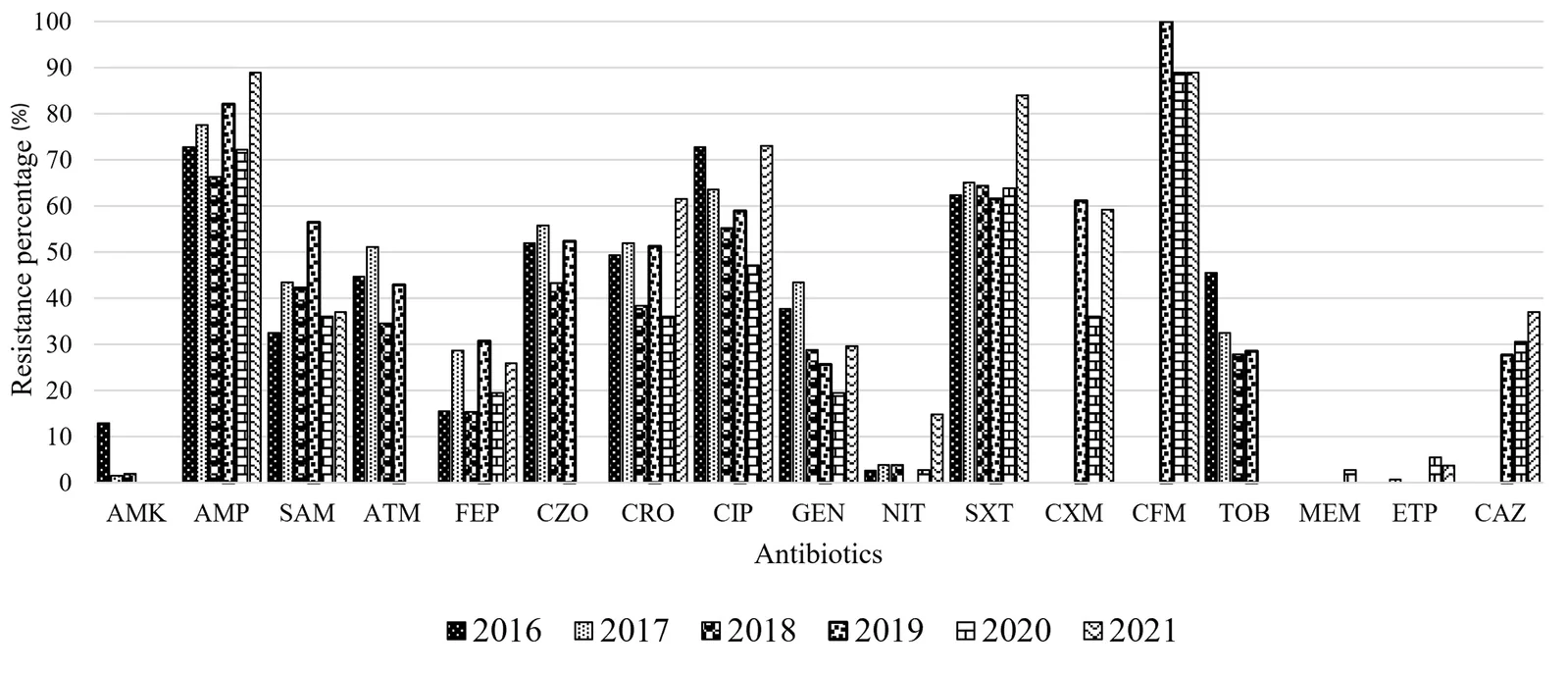
Chronological distribution of AMR Escherichia coli isolates from 2016 to 2021. AMK: amikacin; AMP: ampicillin; SAM: ampicillin/sulbactam; ATM: aztreonam; FEP: cefepime; CZO: cefazolin; CRO: ceftriaxone; CIP: ciprofloxacin; GEN: gentamicin; NIT: nitrofurantoin; SXT: trimethoprim/sulfamethoxazole; CXM: cefuroxime; CFM: cefixime; TOB: tobramycin; MEM: meropenem; ETP: ertapenem; CAZ: ceftazidime; AMR: antimicrobial resistance

A longitudinal analysis of *E. coli* resistance profiles between 2016 and 2021 using the Cochran-Armitage test for linear trend revealed statistically significant linear trends for certain antimicrobial agents (Appendix). A significant downward trend was observed in resistance to GEN (slope = -0.045, p = 0.007), with resistance rates decreasing from 37.7% in 2016 to 19.4% in 2020 (Appendix). In contrast, ETP showed a statistically significant linear upward trend (slope = +0.009, p = 0.007), rising from 0.0% in 2016 to 3.7% in 2021.

For *E. coli*, resistance was also compared between the pre-pandemic period (2016-2019; n = 374) and the COVID-19 pandemic period (2020-2021; n = 57). This analysis identified significant changes in AMR profiles (Table 2), with resistance to MEM being observed during the pandemic period (1.7%), whereas no resistance was detected during the pre-pandemic period (0.0%). A statistically significant increase in resistance to ETP was observed, from 0.2% to 5.2% (Fisher's exact test, p = 0.008). Similarly, AMP increased from 72.4% to 87.7% (χ² = 6.188, p = 0.013), and SXT increased from 63.9% to 80.7% (χ² = 6.346, p = 0.012).

**Table 2:** Comparison of Escherichia coli AMR (%) between the pre-pandemic and pandemic groups. AMP: ampicillin; SAM: ampicillin/sulbactam; FEP: cefepime; CRO: ceftriaxone; CIP: ciprofloxacin; GEN: gentamicin; NIT: nitrofurantoin; SXT: trimethoprim/sulfamethoxazole; ETP: ertapenem; MEM: meropenem; AMR: antimicrobial resistance *χ² value **Fisher’s exact

Antibiotics	Pre-pandemic (n = 374), n (%)	Pandemic (n = 57), n (%)	χ² value or Fisher’s exact	p-value
AMP	271 (72.4%)	50 (87.7%)	6.188	0.013*
SAM	157 (41.9%)	23 (40.3%)	0.046	0.829*
FEP	78 (20.8%)	14 (24.5%)	0.417	0.518*
CRO	172 (45.9%)	30 (52.6%)	0.909	0.340*
CIP	231 (61.7%)	37 (64.9%)	0.230	0.631*
GEN	129 (34.5%)	15 (26.3%)	1.455	0.228*
NIT	12 (3.2%)	5 (8.8%)	Fisher’s exact	0.059**
SXT	239 (63.9%)	46 (80.7%)	6.346	0.012*
ETP	1 (0.2%)	3 (5.2%)	Fisher’s exact	0.008**
MEM	0 (0.0%)	1 (1.7%)	Fisher’s exact	0.132**

Regarding MDR isolates, *E. coli* presented 51.0% MDR isolates, and *K. pneumoniae* showed 32.2% XDR; among less frequently isolated bacteria (n < 30), 100% (n = 24) of MDR isolates were observed in *S. aureus*, and 64.3% MDR and 14.3% possible PDR in *Acinetobacter* spp., and in the case of *P. aeruginosa*, 4.5% possible PDR was observed (Table 3).

**Table 3:** Identification of drug resistance: MDR, XDR, and possible PDR. Data generated via WHONET 2023 (ECDC criteria). ^A^Possible PDR refers to isolates demonstrating non-susceptibility to all tested antimicrobial agents on the local routine AST panel (VITEK® 2, bioMérieux, France). Per ECDC/CDC criteria, definitive PDR status requires testing against all commercially and globally available antimicrobial categories. The percentages corresponding to species with n < 30 (*S. aureus*, *Acinetobacter* spp.) indicate the observed frequency in the sample, but their epidemiological relevance is limited to the evaluated center. MDR: multidrug resistance; XDR: extensive drug resistance; PDR: pandrug resistance; AST: antimicrobial susceptibility testing; ECDC/CDC: European Centre for Disease Prevention and Control/Centers for Disease Control and Prevention

Organism	Isolation number	MDR, n (%)	XDR, n (%)	Possible PDR, n (%)^A^
Escherichia coli	431	220 (51.0%)	41 (9.5%)	--
Klebsiella pneumoniae	143	59 (41.2%)	46 (32.2%)	--
Staphylococcus aureus	24	24 (100%)	--	--
Enterococcus faecalis	44	5 (11.3%)	5 (11.3%)	--
Pseudomonas aeruginosa	44	2 (4.5%)	2 (4.5%)	2 (4.5%)
*Acinetobacter *spp.	14	9 (64.3%)	1 (7.1%)	2 (14.3%)

The comparative analysis of drug resistance between inpatients vs. outpatients (n = 121 vs. n = 898, respectively) focused on species with the highest number of isolates. Higher resistance prevalences were identified in the hospitalized group.

In *E. coli*, significant differences were observed in hospitalized patients for SAM (χ² = 4.422, p = 0.035), TZP (χ² = 17.519, p < 0.001), and CZO (χ² = 6.746, p = 0.009); among inpatients, *E. coli* presented resistance to third-generation cephalosporins greater than 55.0%, as is the case for CRO and CTX, highlighting 100% resistance to CFM. Due to the limited sample size in the inpatient group for *K. pneumoniae* and *P. aeruginosa*, the execution of comparative inferential statistical tests was omitted to avoid bias due to a lack of statistical power. Nevertheless, from a strictly descriptive perspective, 100% resistance to ATM and CZO was identified in *K. pneumoniae* isolates recovered from hospitalized patients. Similarly, for *P. aeruginosa*, resistance levels to TZP and FEP exceeded 60.0% compared to outpatients (Figure 3).

**Figure 3:**
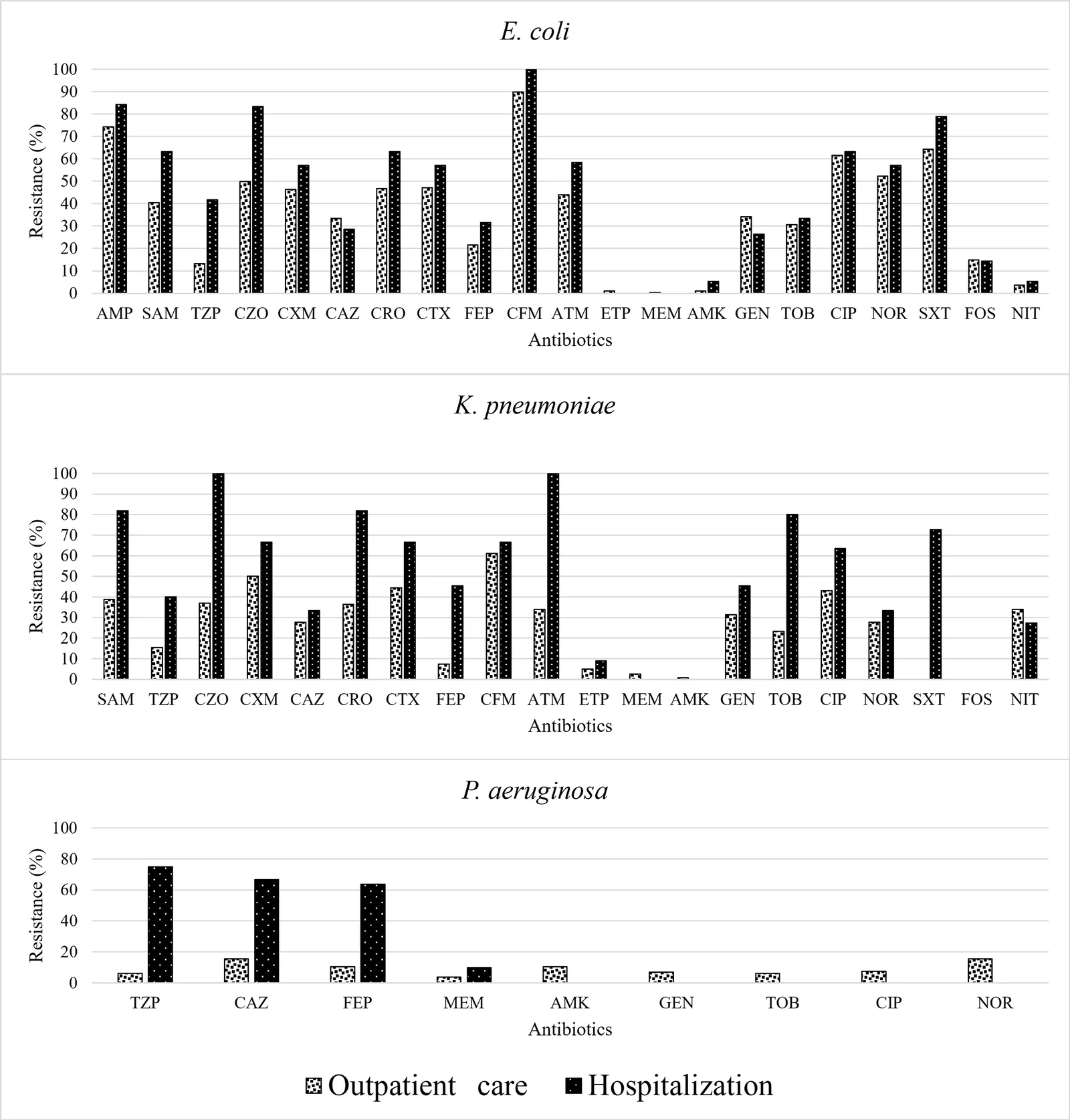
Comparison of antimicrobial resistance in Escherichia coli, Klebsiella pneumoniae, and Pseudomonas aeruginosa isolates from hospitalized patients versus outpatients. N represents 100% of tested isolates for each antimicrobial combination within the outpatient (n = 898) and inpatient (n = 121) cohorts. AMP: ampicillin; SAM: ampicillin/sulbactam; TZP: piperacillin/tazobactam; CZO: cefazolin; CFM: cefixime; CXM: cefuroxime; CAZ: ceftazidime; CTX: cefotaxime; CRO: ceftriaxone; FEP: cefepime; MEM: meropenem; ETP: ertapenem; AMK: amikacin; GEN: gentamicin; TOB: tobramycin; CIP: ciprofloxacin; NOR: norfloxacin; ATM: aztreonam; SXT: trimethoprim/sulfamethoxazole; FOS: fosfomycin; NIT: nitrofurantoin

## Discussion

The COVID-19 pandemic period coincided with marked disruptions in healthcare systems worldwide, as resource reallocation and altered healthcare-seeking behaviors occurred. The vulnerability of individuals exposed to both cancer and COVID-19 may have influenced clinical dynamics in cancer care [22]. The results show a predominant burden of Gram-negative bacilli (73.0%); *E. coli* (42.3%) and *K. pneumoniae* (13.9%) were the most common pathogens. These results are consistent with recent epidemiological reports from Latin America, where Enterobacteriaceae continue to displace Gram-positive bacteria in the oncology setting due to their adaptability and resistance mechanisms; in similar studies, Gram-negative bacteria have consistently been the most prevalent [13,23-25]. Furthermore, several studies have identified *E. coli* [13,14,26,27] and *K. pneumoniae* [5,27,28] as the most prevalent bacteria in immunocompromised patients and in bacterial infections among cancer patients, which is consistent with the results obtained in the present study.

The prevalence of ESBL-producing bacteria (22.6%) is concentrated in *E. coli* (80.0%, n = 184) and *K. pneumoniae* (20.0%, n = 46); however, percentages of up to 40.0% have been reported in other cancer patient populations [14], and up to 31.3% in patients with gastrointestinal diseases [5]. These isolates in cancer patients may limit the effectiveness of drug therapy and increase the length of hospital stay.

When evaluating multiyear resistance trajectories in *E. coli* across the entire 2016-2021 period, temporal fluctuations must be interpreted within a broader observational framework rather than as definitive longitudinal shifts. While short-term comparisons between 2020 and 2021 showed localized increases for CIP and CRO (χ² = 4.144, p = 0.042 and χ² = 4.100, p = 0.043, respectively), formal linear trend analysis across the full six-year timeline demonstrated that overall resistance profiles remained largely stable for most antimicrobial classes. Notably, a statistically significant downward linear trend was identified for GEN (slope = -0.045, p = 0.007), whereas ETP exhibited a small but statistically significant upward linear trend (slope = +0.009, p = 0.007). Short-term variations observed during the 2020-2021 pandemic period must be interpreted as temporal associations rather than direct causal effects of pandemic-era antibiotic administration. These pattern shifts likely reflect unmeasured system-level confounders, including alterations in empirical prescribing behaviors, changes in hospital admission criteria, altered sampling patterns, and temporary disruptions in infection control protocols during the health crisis rather than a permanent biological shift [28]. Nevertheless, third-generation cephalosporin resistance maintained sustained high levels throughout the study period, aligning with global reports documenting widespread resistance in oncology settings [26-28].

Furthermore, it is worth noting the variation in AMP resistance observed in *E. coli* between 2018 and 2021, which ranged from 66.34% to values exceeding 80% at the end of the study (χ² = 4.777, p < 0.05); other studies have reported resistance rates of up to 100% over the past decade [29]; for example, Raraz-Vidal et al. in 2021 [30] reported a 93.9% resistance to AMP in urine samples, while Najera Bello et al. [27] reported a resistance rate of 87.4% across all samples from 620 patients; Sosa Flores et al. [31], in 2022, reported an AMP resistance rate of 88.8% in urine cultures from hospitalized patients, including cancer patients, and in ESBL-producing *E. coli*, 100% resistance to AMP, CXM, and CTX and over 80.0% resistance to CZO, CRO, FEP, and CAZ, indicating that, for this microorganism, penicillins and cephalosporins are losing their efficacy. AMP resistance exceeding 80.0% suggests limited suitability of empirical AMP monotherapy for infections caused by *E. coli* in this local oncology population, highlighting the necessity of relying on individual susceptibility results and current clinical guidelines, consistent with findings described by Sosa Flores et al. [31]. However, despite the high resistance rates observed among penicillins and cephalosporins, carbapenems retained excellent in vitro activity, maintaining 98.0% to 100% susceptibility in *E. coli* (MEM resistance 0.0%-1.7% and ETP resistance 0.2%-5.2%). These findings indicate that carbapenems retained high in vitro activity against *E. coli* isolates in this oncological cohort.

All *S. aureus* isolates (n = 24) were MDR, of which 41.66% were isolated from hospitalized pediatric patients. *S. aureus* is characterized as one of the leading causes of nosocomial bacteremia; a characteristic of this pathogen is its ability to form biofilms, which triggers the production and degradation of organic matter, leading to infection; furthermore, its ability to colonize medical devices such as catheters and heart valves increases the risk of bacteremia or sepsis [32].

Regarding resistance profiles, *K. pneumoniae* stood out as the pathogen with the highest proportion of extensive resistance, with 32.2% of XDR isolates, which is higher than that of *E. coli* (9.5%). This elevated XDR rate is consistent with national surveillance data from Mexican networks such as the University Plan for the Control of Antimicrobial Resistance (PUCRA) [33] as well as regional studies in Latin America [13,23-25], which consistently identify *K. pneumoniae* as a critical reservoir for MDR and XDR phenotypes in tertiary and oncology centers. Globally and regionally, this microorganism has been reported to exhibit resistance rates of up to 60.0% to antibiotics such as GEN, ATM, CIP, TCY, and third- and fourth-generation cephalosporins, demonstrating the decline in the efficacy of antibacterial drugs over the years [34]; however, it has also been reported that *E. coli* exhibits XDR to combinations of beta-lactamase inhibitors, due to the possible production of metallo-beta-lactamases [35].

This ability of *K. pneumoniae* to accumulate resistance genes, particularly against carbapenems and combinations with inhibitors, represents one of the current therapeutic challenges for cancer patients [35]. The marked disparity in drug resistance between outpatients and hospitalized patients is striking. Specifically, resistance to TZP in *E. coli* showed a concerning quantitative increase, rising from 13.0% in the outpatient setting (n = 403) to 42.0% in hospitalized patients (n = 28). A similar pattern was observed in *P. aeruginosa* isolates (n = 12 hospitalized patients vs. n = 32 outpatients). However, this observed disparity must be interpreted with caution and should not be construed as a direct causal effect of hospitalization alone. Instead, these differences are inherently confounded by baseline patient selection bias, where hospitalized oncology patients typically present with greater disease severity, longer hospital stays, distinct age distributions, and prior exposure to broad-spectrum antimicrobials. These results are closely consistent with national scientific evidence; Chavolla-Canal et al. [36] documented resistance over an eight-year period at a secondary-level hospital in Mexico, finding high prevalences of XDR bacteria such as *Pseudomonas*, with a prevalence of 86.4% (n = 38). Furthermore, multiyear surveillance studies in Mexico, such as those by Chavolla-Canal et al. and Ibarra et al., confirm that these non-fermenting bacilli remain among the most resistant isolates in urine cultures [36,37].

Likewise, reports from the PUCRA Network (2024) of the National Autonomous University of Mexico confirm that, although TZP remains a cornerstone in the treatment of Gram-negative bacterial infections in Mexico, resistance in nosocomial *E. coli* has increased due to the coexistence of ESBL-type resistance mechanisms [33], which corroborates the observation made in the present study of a resistance rate of nearly 42.0% in the group of hospitalized patients. This epidemiological gap underscores that empirical management in cancer patients must be tailored according to their setting (community vs. hospital), given the likelihood of treatment failure with TZP in hospitalized patients.

On the other hand, although MDR profiles were identified in *S. aureus* (100%, n = 24) and possible PDR profiles in *Acinetobacter* spp. (14.3%, n = 2/14), these findings should be interpreted with caution. Although their virulence and ability to form biofilms make them microorganisms of great concern [35,38], the low isolation rate in this study limits their epidemiological significance compared to Enterobacteriaceae. The presence of possible PDR isolates in *Acinetobacter* spp. and *P. aeruginosa*, although low in absolute numbers, is consistent with national reports that identify non-fermenting bacilli as the most extreme reservoirs of resistance in intensive care units and pediatric oncology units [26].

When interpreting the findings of the present study, its limitations should be considered. First, due to the use of de-identified routine laboratory surveillance data and patient information protection requirements, it was not possible to evaluate other clinical variables, such as specific tumor type, disease stage, neutropenia status (absolute neutrophil count), and prior individual antibiotic exposure. Second, because urine samples accounted for the vast majority of isolates (79.3%, n = 808), the observed resistance profiles primarily reflect the epidemiology of isolates from urine samples and should not be directly generalized to systemic, bloodstream, or lower respiratory tract infections. Third, comparisons between hospitalized and outpatient patients are subject to inherent selection bias and unmeasured clinical factors; the higher resistance rates observed among hospitalized patients may be influenced by greater disease severity, prolonged hospital stays, or prior exposure to broad-spectrum antimicrobials. Fourth, given the retrospective observational nature of the dataset, the variations in resistance observed during the 2020-2021 period may represent temporal associations rather than direct causal effects related to the management of the COVID-19 pandemic. Finally, the estimation of MDR, XDR, and possible PDR profiles for microorganisms such as *S. aureus* and *Acinetobacter* spp. was limited by the low number of isolates during the study period, which restricted the performance of specific statistical analyses for these microorganisms.

## Conclusions

The analysis of AMR among clinical bacterial isolates from patients with cancer showed a predominance of Enterobacteriaceae, particularly *E. coli* and *K. pneumoniae*. *E. coli* was the most frequently isolated species and exhibited the highest proportion of ESBL production, a high frequency of MDR phenotypes, and low resistance to carbapenems. MDR phenotypes were also identified in *K. pneumoniae*, *E. coli*, *S. aureus*, and *Acinetobacter* spp.; however, the low isolation frequency of the latter two microorganisms limits the epidemiological significance of these findings. Among non-fermenting Gram-negative bacilli, XDR and possible PDR phenotypes were identified. The high resistance rates to third-generation cephalosporins, penicillins, and quinolones, particularly among isolates from hospitalized patients, may limit empirical treatment options and highlight the importance of guiding antimicrobial selection based on local susceptibility profiles.

## Appendices

**Table 4:** Longitudinal antimicrobial resistance trends in Escherichia coli isolates, 2016-2021. nT: total number of isolates; R (%): resistant isolates; S (%): susceptible isolates; AMP: ampicillin; SAM: ampicillin/sulbactam; FEP: cefepime; CRO: ceftriaxone; GEN: gentamicin; NIT: nitrofurantoin; SXT: trimethoprim/sulfamethoxazole; ETP: ertapenem; MEM: meropenem; CIP: ciprofloxacin

Escherichia coli	2016 nT = 80		2017 nT = 150		2018 nT = 105		2019 nT = 39		2020 nT = 36		2021 nT = 21		Pearson chi-squared p ≤ 0.05	Chi-squared (linear trend) p ≤ 0.05	Slope
Antibiotics	R (%)	S (%)	R (%)	S (%)	R (%)	S (%)	R (%)	S (%)	R (%)	S (%)	R (%)	S (%)			
AMP	72.72	25.97	77.52	22.48	66.35	32.69	82.05	17.95	72.22	27.78	88.89	7.41	0.663	0.449	0.011
SAM	32.46	67.53	43.41	56.59	42.31	57.69	56.41	43.59	36.11	63.89	37.04	62.96	0.221	0.422	0.014
FEP	15.58	71.43	28.68	62.02	15.38	75.00	30.77	64.10	19.44	72.22	25.93	62.96	0.070	0.817	0.003
CRO	49.35	50.65	51.94	48.06	38.46	61.54	51.28	48.72	36.11	63.89	61.54	38.46	0.116	0.622	-0.008
CIP	72.73	24.68	63.57	29.46	55.24	36.19	58.97	38.46	47.22	50.00	73.08	15.38	0.038	0.098	-0.028
GEN	37.66	59.74	43.41	56.59	28.85	70.19	25.64	71.79	19.44	80.56	29.63	66.67	0.034	0.007	-0.045
NIT	2.60	90.91	3.88	89.92	3.85	89.42	0.00	92.31	2.78	83.33	14.81	74.07	0.117	0.222	0.008
SXT	62.34	37.66	65.12	34.88	64.42	35.58	61.54	38.46	63.89	36.11	84.00	16.00	0.490	0.276	0.018
ETP	0.00	100.00	0.78	99.22	0.00	100.00	0.00	97.44	5.56	94.44	3.70	96.30	0.016	0.007	0.009
MEM	0.00	100.00	0.00	100.00	0.00	100.00	0.00	100.00	2.78	97.22	0.00	100.00	0.051	0.088	0.002
